# Delta radiomics modeling based on CTP for predicting hemorrhagic transformation after intravenous thrombolysis in acute cerebral infarction: an 8-year retrospective pilot study

**DOI:** 10.3389/fneur.2025.1545631

**Published:** 2025-02-12

**Authors:** Xiaxia Wu, Jinfang Yang, Xianqun Ji, Yingjian Ye, Ping Song, Lina Song, Peng An

**Affiliations:** ^1^Department of Radiology and Surgery, Xiangyang No.1 People’s Hospital, Hubei University of Medicine, Xiangyang, China; ^2^Department of Neurology, NICU and Epidemiology, Xiangyang Key Laboratory of Maternal-Fetal Medicine on Fetal Congenital Heart Disease, Xiangyang No.1 People's Hospital, Hubei University of Medicine, Xiangyang, Hubei, China

**Keywords:** acute cerebral infarction, intravenous thrombolysis, hemorrhagic transformation, CT perfusion imaging, delta radiomics, prediction model

## Abstract

**Objective:**

To explore the value of delta radiomics from cerebral CT perfusion (CTP) in predicting hemorrhagic transformation after intravenous thrombolysis for acute cerebral infarction (HT-ACI).

**Methods:**

Clinical and imaging data of 419 patients with acute cerebral infarction who underwent CTP after treatment between November 2016 and August 2024 were retrospectively collected. Based on post-thrombolysis cranial CT or MRI results, patients were divided into the HT-ACI group (114 cases) and the non-HT-ACI group (305 cases). The dataset was split into a training set and a test set in a 7:3 ratio based on time nodes. In the training set, regions of interest (ROI) within the cerebral infarction area on CTP images were delineated using 3D slicer software, and delta radiomic features were extracted. Hemodynamic parameters such as cerebral blood volume (CBV), cerebral blood flow (CBF), and time to peak (TTP) were obtained using CTP techniques. These were combined with baseline patient data (e.g., age, sex, NIHSS score, medical history) to establish various models for predicting HT-ACI through multivariable logistic regression analysis. The predictive performance of the models was compared using DeLong curves, clinical net benefit was assessed using decision curves, and model predictions were validated using the XGboost algorithm. These results were then validated in the test set, and a nomogram and calibration curve were constructed for clinical application.

**Results:**

In the training set, significant differences were observed between the two groups in NIHSS score, pre-illness usually use of anticoagulants, age, infarction size, ADC difference, CBF, and Delta radscore (*P* < 0.05). The combined model [AUC 0.878, OR 0.0217, 95%CI 0.835–0.913] demonstrated superior predictive performance compared to the clinical model [AUC 0.725, OR 0.0310, 95%CI 0.670–0.775] and the imaging model [AUC 0.818, OR 0.0259, 95%CI 0.769–0.861]. This was confirmed by the XGboost algorithm, and decision curves confirmed the higher clinical net benefit of the combined model. Similar results were validated in the test set, and a novel nomogram was constructed to simplify the prediction process for HT-ACI.

**Conclusion:**

The combined model established based on delta radiomics from CTP may provide early insights into the hemodynamic status of acutely ischemic brain tissue, holding significant clinical importance for predicting HT-ACI. This method could offer a powerful imaging reference for clinical decision-making in patients with ACI, helping to reduce the risk of HT-ACI and improve patient outcomes.

## Introduction

Acute cerebral infarction, characterized by high incidence, disability, and mortality rates, remains a significant clinical challenge. The prognosis of this disease is poor, especially when complicated by hemorrhagic transformation after intravenous thrombolysis for acute cerebral infarction (HT-ACI). HT-ACI occurs due to secondary hemorrhage resulting from blood–brain barrier (BBB) disruption and reperfusion injury in ischemic brain tissue following acute cerebral infarction. This process can occur at any stage of the natural course of acute cerebral infarction or after stroke treatment. Statistics indicate that the incidence of HT-ACI is as high as 10–20%, with 30 to 40% being progressive, profoundly impacting patients’ treatment plans and prognosis (1–4). Given the severe consequences of HT-ACI, clinicians urgently need a method to predict the risk of post-treatment rebleeding in patients early, thereby enhancing the safety of endovascular treatment and improving patient outcomes. However, traditional assessment methods based on clinical symptoms and conventional imaging features have certain limitations. Clinical symptom evaluation is often subjective and lacks accuracy, while conventional imaging findings cannot provide timely and accurate diagnoses. Therefore, identifying sensitive predictive markers for HT-ACI is crucial (5–7). In recent years, the rapid development of radiomics has offered new opportunities for HT-ACI prediction. By mining and screening a vast amount of quantitative feature information from acute cerebral infarction images, radiomics can establish classifiers to aid in the judgment of HT-ACI occurrence, enabling precise diagnosis and prediction. This approach is expected to overcome the limitations of traditional assessment methods and improve prediction accuracy. Currently, some studies have effectively predicted the occurrence of HT-ACI using radiomics combined with machine learning models or artificial intelligence, but these are all based on static radiomics, that is, radiomic parameters extracted from non-contrast head CT scans, which may introduce certain biases (8–10). Therefore, this study aims to construct a model for predicting HT-ACI based on delta radiomic features derived from cerebral CT perfusion (CTP) images. This not only contributes to a deeper understanding of the pathogenesis of HT-ACI but also provides clinicians with a novel and more accurate predictive tool to guide clinical treatment decisions and improve patient prognosis.

## Materials and methods

### Study materials

**Figure 1:**
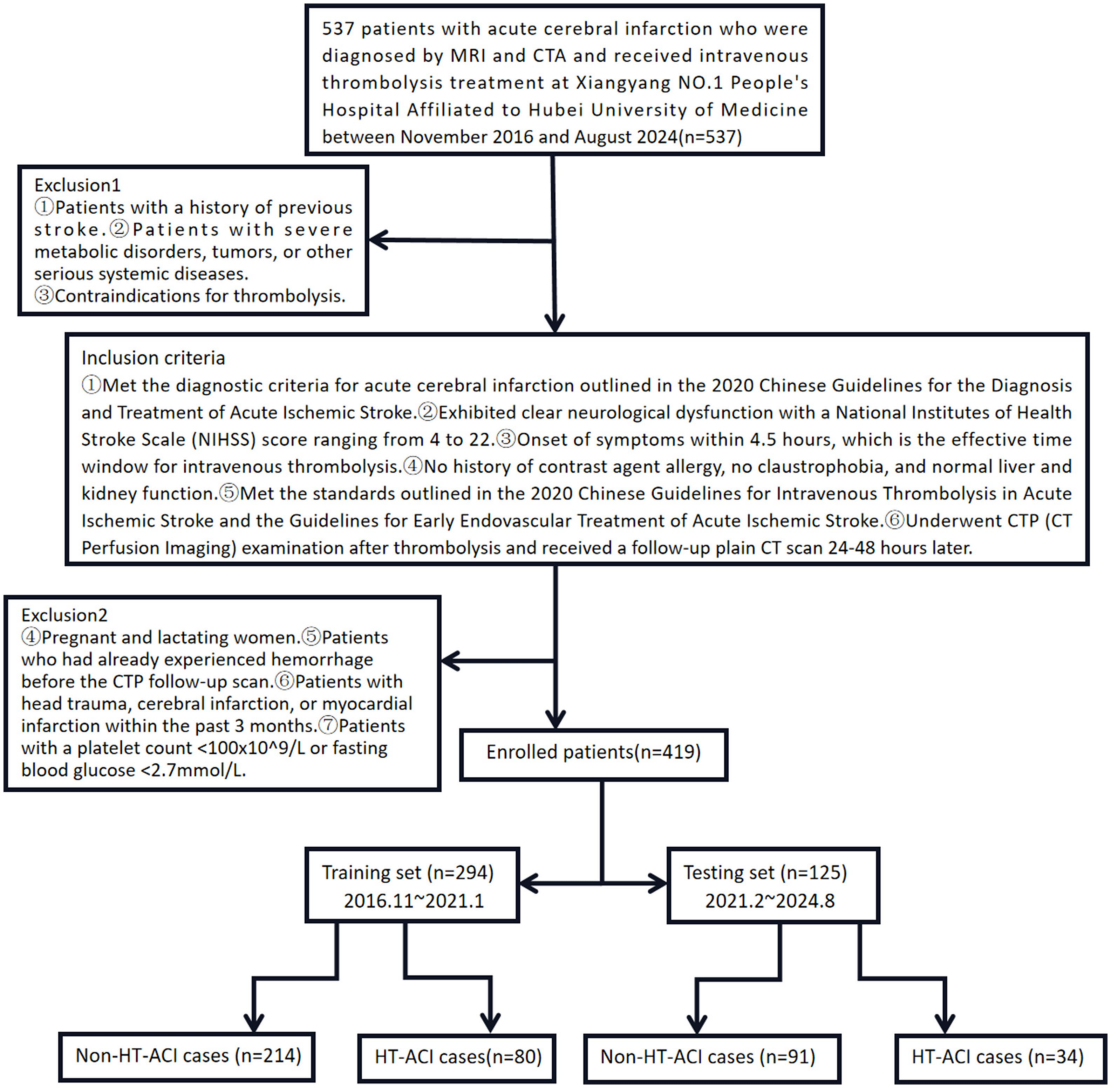
Inclusion and exclusion criteria, and case grouping method for this study.

We retrospectively collected and analyzed clinical and imaging data from 537 patients with acute cerebral infarction who were diagnosed by MRI and CTA and received intravenous thrombolysis treatment at Xiangyang NO.1 People’s Hospital Affiliated to Hubei University of Medicine between November 2016 and August 2024. Inclusion Criteria: ① Met the diagnostic criteria for acute cerebral infarction outlined in the 2020 Chinese Guidelines for the Diagnosis and Treatment of Acute Ischemic Stroke. ② Exhibited clear neurological dysfunction with a National Institutes of Health Stroke Scale (NIHSS) score ranging from 4 to 22. ③ Onset of symptoms within 4.5 h, which is the effective time window for intravenous thrombolysis. ④ No history of contrast agent allergy, no claustrophobia, and normal liver and kidney function. ⑤ Met the standards outlined in the 2020 Chinese Guidelines for Intravenous Thrombolysis in Acute Ischemic Stroke and the Guidelines for Early Endovascular Treatment of Acute Ischemic Stroke. ⑥ Underwent CTP (CT Perfusion Imaging) examination after thrombolysis and received a follow-up plain CT scan 24–48 h later. Exclusion Criteria: ① Patients with a history of previous stroke. ② Patients with severe metabolic disorders, tumors, or other serious systemic diseases. ③ Contraindications for thrombolysis. ④ Pregnant and lactating women. ⑤ Patients who had already experienced hemorrhage before the CTP follow-up scan. ⑥ Patients with head trauma, cerebral infarction, or myocardial infarction within the past 3 months. ⑦ Patients with a platelet count <100×10^9/L or fasting blood glucose <2.7 mmol/L (11, 12). Final Enrollment: After rigorous screening and exclusion, 419 patients were enrolled, including 234 males and 185 females, aged between 36 and 85 years, with an average age of (57.6 ± 18.5) years. The patients were closely followed up for their disease outcomes. Based on CT or MRI results, patients were divided into the HT-ACI group (114 patients) and the non-HT-ACI group (305 patients) (Figure 1).

### Examination methods

Equipment: Siemens SOMATOM Drive Dual-Source 64-Slice CT scanners were used. Patient Position: Patients were placed in a supine position on the examination table. Scanning Baseline: The scanning baseline was set at the orbitomeatal line. Plain Scan Parameters: Tube voltage: 120 kV, tube current: 300 mA, slice thickness: 3 mm, slice interval: 1 mm. CTP Scan: Based on the plain CT scan results, a total of 23 slices covering the whole brain were determined. Iopromide contrast agent (350 mg/mL) was injected as a bolus through the right elbow vein using a high-pressure injector at a rate of 4.5 mL/s. Scanning began 5 s after the injection. Scanning Parameters: Tube voltage: 80 kV, tube current: 200 mAs, matrix: 512×512, slice thickness: 1 mm, slice interval: 1, interval: 2 s, continuous scanning for 45 s, scanning range: 200 mm. A total of 700–900 frames of images were obtained. CTP Image Processing and Data Analysis, Image Transfer: The reconstructed dynamic images were transferred to the GE ADW4.6 workstation. Software Processing: The BRAIN STROKE Perfusion software (non-deconvolution/deconvolution algorithm) was used for image post-processing. Data Obtained: Time-intensity curves, CT perfusion images, CBF maps, CBV maps, and TTP maps of the region of interest (ROI). Image Analysis: Two senior imaging specialists with over 10 years of experience analyzed the images using a double-blind method. The area centered on the infarct lesion was designated as the ROI, avoiding blood vessels and sulci of the brain. The absolute values of CBF, CBV, and TTP in the infarcted side and the corresponding contralateral region were calculated (13, 14). ADC Difference: The lowest ADC value (mm^2^/s) in the core of the infarct lesion and the ADC value (mm^2^/s) in the contralateral mirror region were measured on the ADC sequence of brain MRI, and the ADC difference between the contralateral and infarcted sides was calculated.

### Image segmentation and radiomic feature extraction in cerebral infarct regions

**Figure 2:**
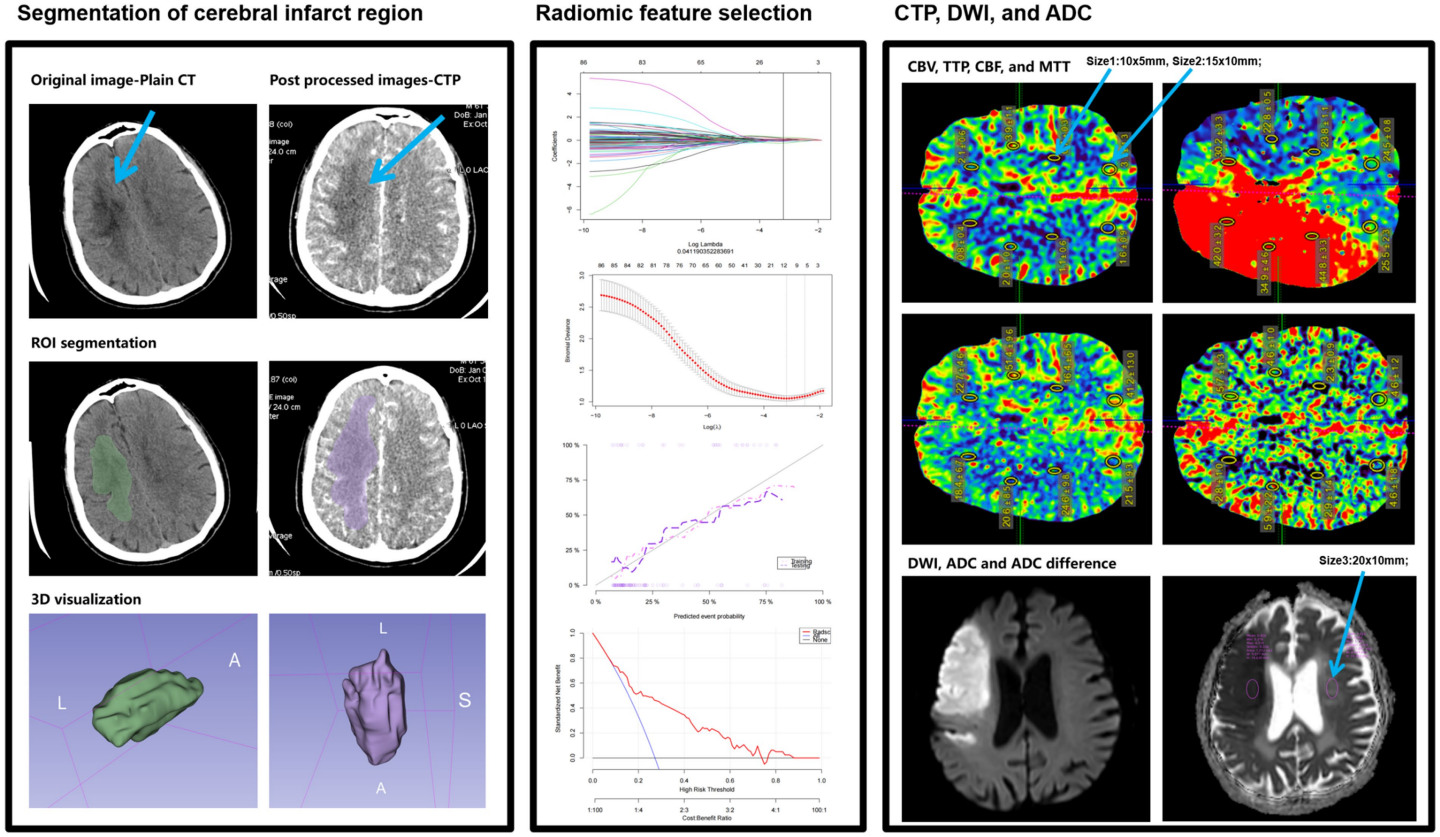
A simple schematic diagram of ROI delineation and radiomic feature extraction in cerebral infarct regions in this study.

Using the 3D slicer software (version 4.11), the region of interest (ROI) for the entire cerebral infarct region was segmented layer by layer on 1.0 mm thin-slice CTP images. The criteria for identifying the infarct region were defined as follows: based on a comprehensive assessment of the DWI sequence from MR and plain CT scans, the entire infarct region on CTP was delineated as the ROI. The ROI for the infarct region encompassed the core infarct area and the ischemic penumbra within 3–10 mm around it, excluding surrounding cerebral sulci, cisterns, subarachnoid spaces, and bone tissue. After manually delineating the ROI for the infarct region layer by layer, revisions were made with reference to coronal and sagittal MR images. The segmentation process of the infarct region was manually conducted by two radiologists with over 10 years of experience in neurological radiology, and the intraclass correlation coefficient (ICC) was used to evaluate the consistency of segmentation parameters and the reproducibility of radiomic feature extraction between the two radiologists. An ICC > 0.75 indicated good consistency and reproducibility of radiomic feature extraction. The Radiomics plugin in Slicer was utilized to extract radiomic features, with image resampling and nearest-neighbor interpolation performed prior to this to standardize the CTP images. A total of 879 × 2 radiomic features were extracted, including shape features, first-order statistical features, texture features, and higher-order features. Subsequently, the LASSO algorithm and 10-fold cross-validation were employed to generate the Radscore based on the remaining features; Radscore1 represented the radiomic score derived from texture parameters extracted from plain CT scans, while Delta Radscore represented the radiomic score calculated as (CTP cerebral perfusion phase texture parameters – plain CT scan phase texture parameters)/CTP cerebral perfusion phase texture parameters (15) (Figure 2).

### Overview of statistical methods

Statistical analysis was conducted using SPSS 22.0 and R software. Firstly, the enrolled cases were divided into a training set and a test set based on a 7:3 ratio and time points. All continuous variables underwent tests for homogeneity of variance and normal distribution. Measurement data for the two groups (normally distributed) were expressed as X ± S and compared using *t*-tests; measurement data for the two groups (non-normally distributed) were expressed as ranges (median, 25–75%) and compared using rank-sum tests. Categorical variables were expressed as percentages (%) or specific case numbers and compared using chi-square tests or Fisher’s exact tests. For radiomic parameters, the LASSO algorithm and 10-fold cross-validation were used to adjust elastic network parameters to avoid overfitting, thereby selecting the optimal texture features to generate the radiomic score (Radscore). In the training set, three prediction models (clinical model, imaging model, and combined model) were established using multivariate logistic regression. The predictive performance of the models was compared using DeLong’s test, and the clinical net benefit of the most valuable model was assessed using decision curves. These results were then validated in the test set and further verified using the XGboost machine learning model by outputting SHAP values. Finally, a novel nomogram was established and corresponding clinical trials and applications were conducted. A *p*-value <0.05 indicated statistical significance (16).

## Results

A total of 537 patients with acute ischemic stroke caused by anterior circulation vascular occlusion underwent non-contrast CT, CTP, and CTA examinations, with the time from stroke onset to CT imaging being ≤6 h were enrolled. One hundred and eighteen patients were excluded due to reasons such as poor image quality or motion artifacts, presence of old cerebral infarction, lack of follow-up imaging data, contrast agent extravasation, occurrence of hemorrhage before CTP reexamination, and patients with cerebral infarction or myocardial infarction. Ultimately, 419 patients met the study criteria, with an average age of (57.6 ± 18.5) years. Univariate regression analysis revealed no statistically significant differences between the two patient groups in terms of gender, history of coronary heart disease, history of atrial fibrillation, previous stroke or transient ischemic attack (TIA), history of hypertension, history of diabetes, smoking history, alcohol consumption history, prothrombin time (PT), body mass index (BMI), infarct location, pulmonary infection (PI), white blood cell count, D-dimer levels, platelet-to-lymphocyte ratio (PLR), neutrophil-to-lymphocyte ratio (NLR), cerebral blood volume (CBV), time to peak (TTP), and Radscore 1 (*p* > 0.05). Significant statistical differences were observed between the two groups in infarct size, National Institutes of Health Stroke Scale (NIHSS) score, pre-illness regular use of anticoagulants, age, cerebral blood flow (CBF), ADC difference, and Delta Radscore (*p* < 0.05). Multivariate regression analysis confirmed that infarct size, age, CBF, and Delta Radscore were independent risk factors for the occurrence of hemorrhagic transformation after acute cerebral infarction (HT-ACI).

**Figure 3:**
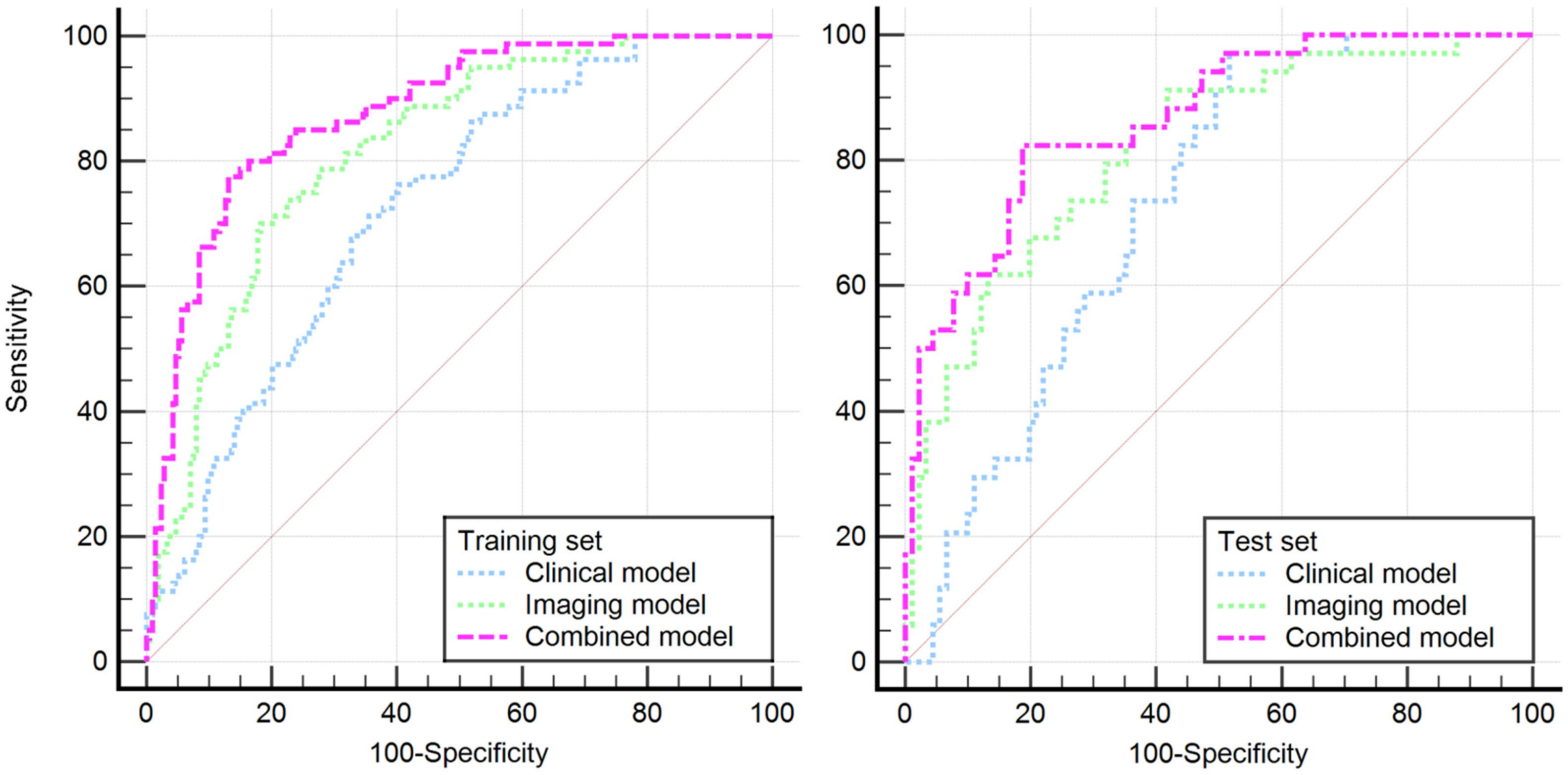
Illustrates the DeLong’s non-parametric curves for both the training and test sets, revealing that the combined model has the largest area under the ROC curve, confirming its optimal predictive performance.

**Figure 4:**
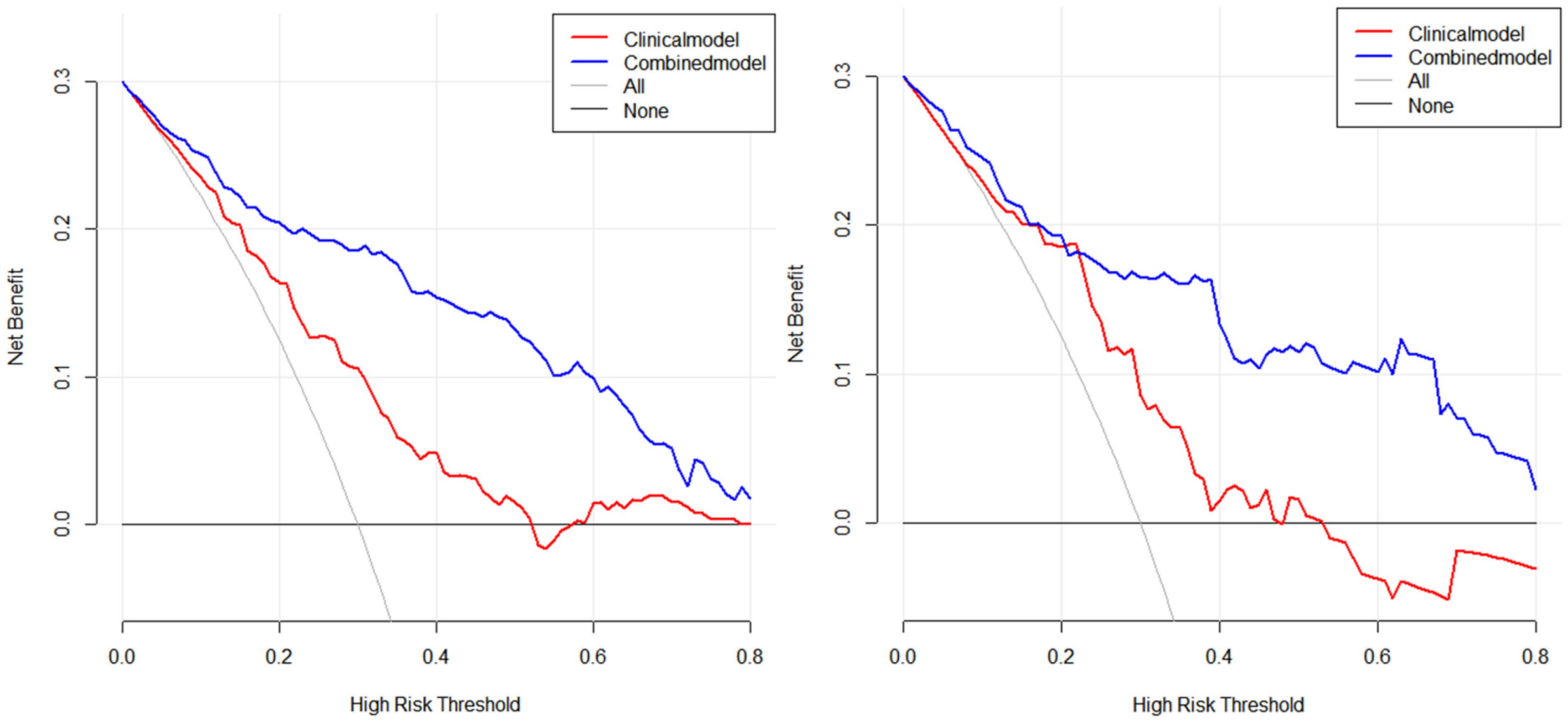
Presents the DCA analysis conducted using R software for both the training and test sets, further confirming the higher clinical net benefit of the combined model (left: training set, right: test set).

**Figure 5:**
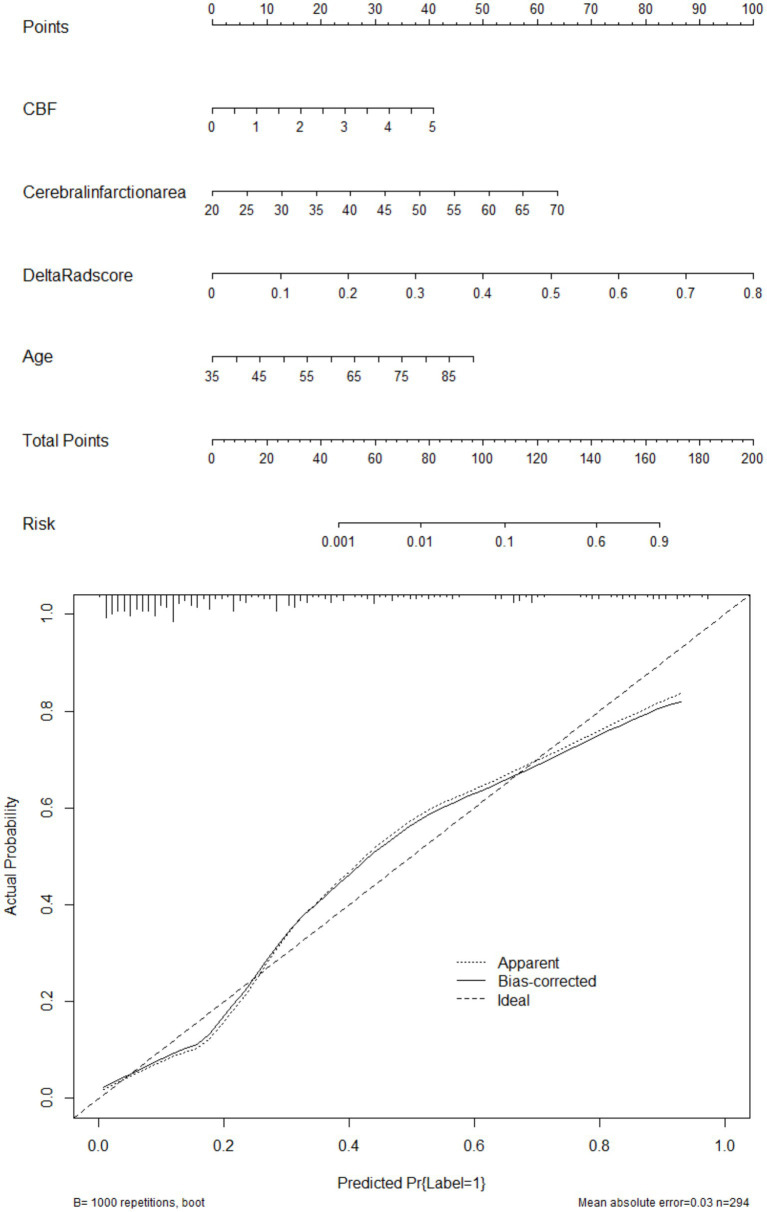
Showcases the clinically well-regarded nomogram prediction tool based on risk factors from the combined model (above: nomogram, below: calibration curve). This tool simplifies the assessment process for HT-ACI by assigning scores to each risk factor and summing them up to calculate the final risk value. For example, in Case 501, the CBF of 4.5 corresponds to 37.5 points, the infarct area of 40 corresponds to 25 points, the Delta Radscore of 0.6 corresponds to 75 points, and the age of 70 corresponds to 37.5 points, totaling 175 points, which corresponds to a risk value >0.9. It is inferred that this patient has a high probability of developing HT-ACI, consistent with the clinical outcome.

**Figure 6:**
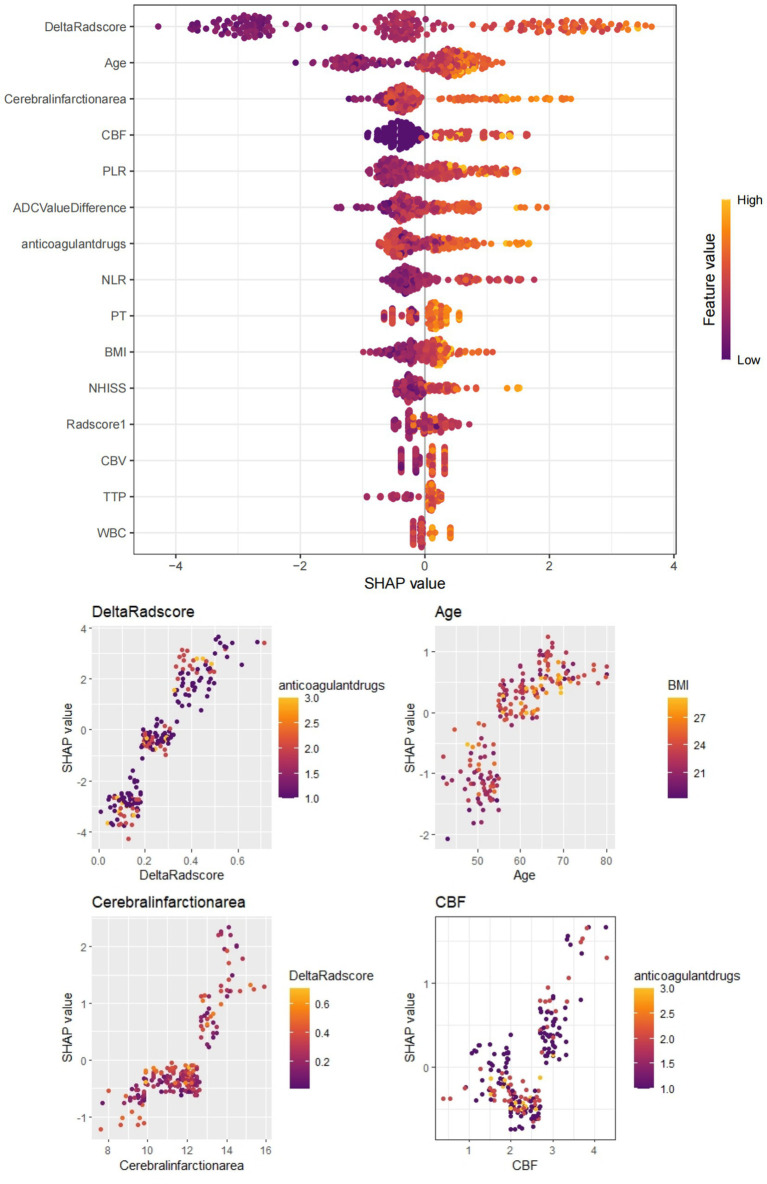
Depicts the SHAP value plot output by the XGBoost machine learning model. The SHAP values confirm that infarct size (cut-off value 51.30), age (55.81), CBF (3.12), Delta Radscore (0.31), etc., are important factors influencing the occurrence of HT-ACI, aligning with our research findings. Red represents lower values, while yellow represents higher values.

Based on these risk factors, we established clinical, imaging, and combined models. In the training set, the combined model demonstrated the highest predictive performance [AUC 0.878, OR 0.0217, 95% CI 0.835–0.913, sensitivity 86.92%, specificity 77.51%], significantly outperforming the clinical model [AUC 0.725, OR 0.0310, 95% CI 0.670–0.775, sensitivity 72.15%, specificity 60.81%, *p* < 0.05] and the imaging model [AUC 0.818, OR 0.0259, 95% CI 0.769–0.861, sensitivity 81.70%, specificity 70.15%, *p* < 0.05]. Similar results were validated in the test set, with the combined model showing superior predictive performance [AUC: 0.867, OR 0.0353, 95% CI: 0.794–0.921, sensitivity 82.35%, specificity 81.32%] compared to the clinical model [AUC: 0.729, OR 0.0448, 95% CI: 0.642–0.804, sensitivity 69.51%, specificity 71.32%, *p* = 0.001] and the imaging model [AUC: 0.818, OR 0.0422, 95% CI: 0.739–0.882, sensitivity 80.61%, specificity 75.19%, *p* = 0.04]. Decision curve analysis (DCA) confirmed that the combined model had higher clinical net benefit in both groups, and the XGboost machine learning model also verified the association of infarct size, age, CBF, and Delta Radscore with the occurrence of HT-ACI (all *p* < 0.05). Subsequently, a nomogram and calibration curve based on the combined model were developed and received clinical application and positive feedback (Tables 1–3 and Figures 3–6).

**Table 1:** Presents the logistic regression analysis results of the clinical model predicting HT-ACI based on clinical features, with *p < 0.05 indicating statistical significance.

Clinical model	Univariate analysis		Multivariate analysis	
Factors	*P*	Hazard ratio	*P*	Hazard ratio
Gender	0.36	1.27 (0.76–2.10)		
History of coronary heart disease	0.69	1.14 (0.58–2.22)		
History of atrial fibrillation	0.41	1.27 (0.71–2.24)		
Previous stroke or transient ischemic attack (TIA)	0.13	1.35 (0.91–1.98)		
History of hypertension	0.54	1.01 (0.97–1.05)		
History of diabetes	0.13	1.04 (0.98–1.09)		
Smoking history	0.37	1.02 (0.98–1.05)		
Alcohol consumption history	0.15	1.02 (0.99–1.04)		
Prothrombin time (PT)	0.12	1.15 (0.96–1.38)		
BMI	0.13	1.09 (0.97–1.20)		
Infarct location	0.34	1.15 (0.86–1.54)		
Pulmonary infection (PI)	0.38	1.18 (0.82–1.72)		
White blood cell count	0.19	1.09 (0.96–1.24)		
D-dimer levels	0.61	0.94 (0.77–1.17)		
PLR	0.11	1.04 (0.99–1.09)		
NLR	0.12	1.05 (1.98–1.10)		
Infarct size	<0.05*	1.09 (1.05–1.13)	<0.05*	1.09 (1.04–1.12)
NIHSS	<0.05*	1.28 (1.12–1.48)	0.01*	1.24 (1.07–1.43)
Pre-illness regular use of anticoagulants	0.01*	1.68 (1.15–2.47)		
Age	0.04*	1.03 (1.01–1.07)		

*Multivariate regression confirmed that the Infarct size and NIHSS were influential factors for HT-ACI.

**Table 2:** Displays the logistic regression analysis results of the imaging model predicting HT-ACI based on imaging features, with *P < 0.05 indicating statistical significance.

Imaging model	Univariate analysis		Multivariate analysis	
Factors	*P*	Hazard ratio	*P*	Hazard ratio
CBF	0.04*	1.49 (1.01–2.23)		
ADC difference	0.04*	1.01 (1.00–1.02)	0.03*	1.01 (1.00–1.02)
CBV	0.43	1.32 (0.66–2.61)		
TTP	0.22	2.84 (0.54–14.92)		
Delta Radscore	<0.05*	2.34 (1.85–2.96)	<0.05*	2.41 (1.89–3.07)
Radscore 1	>0.05			

*Multivariate regression confirmed that the ADC difference and Delta Radscore were influential factors for HT-ACI.

**Table 3:** Shows the logistic regression analysis results of the combined model predicting HT-ACI based on the valuable factors mentioned above, with *P < 0.05 indicating statistical significance.

Combined model	Univariate analysis		Multivariate analysis	
Factors	*P*	Hazard ratio	*P*	Hazard ratio
Infarct size	<0.05*	1.09 (1.05–1.13)	<0.05*	1.09 (1.04–1.15)
NIHSS	<0.05*	1.28 (1.12–1.48)		
Pre-illness regular use of anticoagulants	0.01*	1.68 (1.15–2.47)		
Age	0.04*	1.03 (1.01–1.07)	<0.05*	1.06 (1.02–1.11)
CBF	0.04*	1.49 (1.01–2.23)	<0.05*	1.92 (1.14–3.24)
ADC difference	0.04*	1.01 (1.00–1.02)		
Delta radscore	<0.05*	2.34 (1.85–2.96)	<0.05*	2.66 (2.02–3.51)

*Multivariate regression confirmed that the Infarct size, age, CBF and Delta Radscore were influential factors for HT-ACI.

## Discussion

With the intensification of domestic environmental pollution and the aggravation of social aging, the incidence rate of stroke has reached 200/100,000, with ischemic stroke accounting for 69.6% of all strokes. Intravenous thrombolysis is currently one of the most effective treatments for acute cerebral infarction. Hemorrhagic transformation after acute cerebral infarction (HT-ACI) refers to secondary hemorrhage within the cerebral infarction area or hemorrhage in adjacent areas due to the restoration of blood flow perfusion in the ischemic area of the brain after cerebral infarction. HT-ACI is a serious complication of intravenous thrombolysis, significantly affecting its therapeutic effect. It is also one of the main reasons for neurological deterioration in patients with cerebral infarction after intravenous thrombolysis and for medical disputes. Previous studies have suggested that low serum albumin levels, smoking history, diabetes mellitus, coagulation time, and CTP parameters [cerebral blood volume (CBV), time to peak (TTP), mean transit time (MTT)] are associated with poor prognosis in patients with stroke, but these factors lack specificity (17–19). Furthermore, conventional CT and MR have certain difficulties and limitations in predicting HT-ACI, mainly due to the complexity of HT-ACI, limitations of imaging techniques, and individual differences among patients. The mechanisms of HT-ACI involve multiple aspects, including post-infarction ischemic injury, reperfusion injury, coagulation dysfunction, and blood–brain barrier disruption. These factors interact with each other, making the occurrence of HT-ACI highly complex and uncertain. Restricted by the examination time window, CT and MR have low sensitivity to small or micro-hemorrhage points, which are difficult to distinguish from surrounding brain tissue, potentially leading to delayed detection of HT-ACI and exacerbation of the condition (20, 21). Our team has found that radiomic parameters based on delta changes in cerebral infarction areas, extracted from enhanced CT cerebral perfusion imaging and plain CT, are helpful in predicting HT-ACI. Therefore, this study established a novel nomogram for predicting HT-ACI based on clinical parameters combined with the Delta radscore, which has received favorable clinical evaluations. This nomogram is non-invasive and requires no additional costs, with a high AUC value of 0.878, providing a new method for the prediction and treatment evaluation of HT-ACI.

This study found that HT-ACI is correlated with certain factors, including the NIHSS score, pre-illness usually use of anticoagulants, age, infarct size, ADC difference, CBF, and Delta radscore. The NIHSS score (National Institutes of Health Stroke Scale score) is a crucial indicator for assessing stroke severity. Research has shown that the NIHSS score is closely related to the risk of HT-ACI, with a higher NIHSS score generally indicating a higher risk of HT-ACI. It has been reported that patients with an NIHSS score ≥ 20 have an increased risk of fatal hemorrhagic transformation, reaching 6.8%. The routine use of anticoagulants (such as Warfarin, Clopidogrel Sulfate, Aspirin, etc.) before illness also increases the risk of HT-ACI. Anticoagulants prevent thrombus formation by inhibiting the coagulation process but simultaneously increase the risk of bleeding. When administering intravenous thrombolysis to patients using anticoagulants, it is necessary to more cautiously assess the risk of HT-ACI and closely monitor the patient’s bleeding status. Age is an important risk factor for HT-ACI. As age increases, the blood–brain barrier becomes relatively fragile, and the elasticity and toughness of blood vessel walls gradually decrease, making them more prone to rupture and bleeding. Therefore, the risk of HT-ACI significantly increases in elderly patients undergoing intravenous thrombolysis. Generally, the larger the infarct size, the higher the risk of HT-ACI. Large cerebral infarcts can lead to significant brain edema, exerting pressure on surrounding capillaries. When reperfusion is restored and collateral circulation opens up, it may cause blood vessels to rupture, resulting in a marked increase in the incidence of HT-ACI. This study confirms that patients with large infarcts (maximum infarct area ≥ 50 cm^2^ or infarct volume ≥ 145 mL) have a sixfold increase in the incidence of HT-ACI compared to patients with smaller infarcts (21, 22). In this study, ADC difference, CBF, and Delta radscore are newly identified predictors of HT-ACI. The ADC (apparent diffusion coefficient) difference reflects differences in water molecule diffusion capacity between the infarct core and normal regions. In this study, the ADC difference in the HT-ACI group was significantly higher than that in the normal group (*p* < 0.05), which may be related to ischemia, hypoxia, vascular damage, and blood–brain barrier disruption caused by acute cerebral infarction. Ongoing ischemia and hypoxia in the brain tissue of patients with acute cerebral infarction can lead to cytotoxic edema, increased DWI signals, and significantly reduced ADC values. The ADC difference is collected and calculated from the mirror position of brain tissue in the same individual, effectively avoiding individual differences caused by factors such as the concentration and viscosity of intracellular and extracellular water and body temperature. This allows for an objective and accurate assessment of the cerebral infarction area, thus aiding in the prediction of HT-ACI. Additionally, we found that when the CBF value exceeds 3.12, the incidence of HT-ACI significantly increases. An overly rapid or excessive restoration of cerebral blood flow can lead to an increase in cerebral reperfusion, potentially elevating the risk of hemorrhagic transformation. This is because the damaged vascular walls, upon the resumption of blood flow, may rupture and bleed due to the intense impact of the blood. Furthermore, the dosage and timing of thrombolytic drugs also affect the restoration of cerebral blood flow and the risk of hemorrhagic transformation. If the dosage of thrombolytic drugs is too high or the timing is inappropriate, it may result in an overly rapid restoration of cerebral blood flow, thereby increasing the risk of HT-ACI (23, 24).

Radiomics is an advanced technique that extracts a large number of features (such as shape, intensity, texture, etc.) from medical imaging data and performs high-throughput analysis. These features can reflect the biological characteristics and malignant progression potential of lesions, providing reliable auxiliary information for disease diagnosis. In the diagnosis and treatment of multisystem tumors, radiomics technology can analyze medical imaging data such as CT and MRI to extract quantitative features related to tumors, thereby assisting doctors in early diagnosis and decision-making. It is reported that prediction models built using radiomics technology can effectively classify four types of brain tumors, including craniopharyngioma, glioma, glioblastoma, and metastasis, with high accuracy demonstrated in various validation sets. Moreover, radiomics models based on machine learning algorithms have shown higher accuracy than traditional imaging methods in differentiating these brain tumors. Therefore, for the prediction of HT-ACI, we have incorporated the relatively novel Delta radscore. This high-quality data, based on perfusion changes in enhanced CT, helps to extract CT grayscale texture changes before and after thrombolysis in the acute cerebral infarction area, providing reliable data to evaluate the degree of blood flow perfusion and cerebral tissue infarction within the infarcted area, and further guiding the treatment of acute cerebral infarction and predicting HT-ACI. In this study, based on CTP (CT Perfusion) images, a total of 879 × 2 radiomic features were extracted from the ROI (Region of Interest) of the whole infarcted area. After screening for redundant features, 859 × 2 radiomic features were ultimately retained, including morphological features such as Sphericity, and high-order texture features such as RunLengthNonUniformity.0.732, Skewness.0.588, and Kurtosis.0.393. Sphericity is a shape feature in radiomics that intuitively reflects the texture features of the image or object surface, describing the shape, roughness, smoothness, and grayscale differences of the tissue. A larger value indicates greater differences, which may be related to tissue ischemia, necrosis, and softening. This study found that acute cerebral infarction patients with lower Sphericity values are more prone to HT-ACI, suggesting that patients with acute cerebral infarction where the infarcted tissue has not completely softened and necrosed should be cautious about the dosage during thrombolysis to avoid excessive perfusion leading to HT-ACI. High-order texture features generated through frequency domain denoising methods such as Fourier transform and wavelet transform emphasize areas of grayscale change and their texture heterogeneity, allowing more valuable radiomic features to be extracted from the original image. RunLengthNonUniformity.0.732 reflects the uniformity or orderliness of grayscale distribution within the image or infarcted ROI, with a smaller value indicating a more uniform and ordered grayscale distribution. Skewness.0.588 and Kurtosis.0.393 measure the difference in grayscale values between adjacent pixels within the image or ROI. A smaller value indicates smaller differences in grayscale values between adjacent pixels, resulting in a smoother image; a larger value indicates larger differences, resulting in a rougher image. Both can more finely assess the grayscale texture differences and perfusion differences between the infarcted area and surrounding tissues, with a quantization degree of tissue differences significantly higher than that of human visual recognition (25–28). Therefore, the combined model based on CTP radiomics has shown good prediction results for HT-ACI, outperforming clinical and imaging models. The combined model can provide strong support for neurologists to develop personalized treatment plans and provide prognostic guidance. Meanwhile, this study employed dynamic radiomics techniques, which offer certain advantages over previous CTP studies. It emphasizes the radiomic texture changes before and after treatment, avoiding the gray areas that are indistinguishable by the naked eye, thus supplementing the deficiencies of previous research and promoting new developments in CTP radiomics. More importantly, this study incorporated machine learning models and used SHAP plots to verify the predictive factors of the nomogram, and conducted corresponding clinical practical applications (29).

### Limitations

Firstly, due to the single-center nature of the study, the sample size remains relatively small, and the study population is primarily Han Chinese, with potential differences in dietary and lifestyle habits. Consequently, biases may inevitably exist in the study results. Future research will involve multi-center and multi-regional studies to validate the conclusions of this study. The use of unsupervised fully or semi-automatic delineation of infarcted regions of interest (ROIs) was not employed, potentially introducing errors. The exclusion of radiomic parameters from MR plain scans and multi-phase enhanced scans may have omitted meaningful variables. Additionally, this study did not utilize radiomics to assess the impact of HT-ACI status on patient prognosis, which may limit or challenge clinical decisions based on these study results. This study focuses on anterior circulation ischemic stroke and does not investigate the value of CTP radiomics in middle-posterior circulation stroke or venous stroke. However, we believe that this research also holds certain value in other subtypes of stroke, and we anticipate future studies with larger sample sizes to validate this conclusion. Although we included patients’ age and gender as calibration factors in our multivariate statistical analysis, and we also tried our best to balance the proportion of positive patients in the training and test sets to enhance the reliability and robustness of this study, differences in sample size are inevitable, which has raised concerns about the generality and reliability of the results. In the future, we plan to expand our sample size to 5,000 cases to reduce the error caused by this imbalance, and we believe that the results of this study hold some significance (30).

## Conclusion

In summary, the Delta radscore has demonstrated significant clinical value in the study of HT-ACI. The novel nomogram established based on the Delta radscore may simplify and effectively predict HT-ACI, aiding in improving treatment decisions for patients with acute cerebral infarction and enhancing perioperative care. With continuous advancements in imaging technology and deepening research, the application of radiomics in the field of cerebral hemorrhage will become increasingly widespread, offering more possibilities for disease prevention, diagnosis, and treatment.

## Data Availability

The original contributions presented in the study are included in the article/supplementary material, further inquiries can be directed to the corresponding authors.
